# Endocytosis of Coacervates into Liposomes

**DOI:** 10.1021/jacs.2c04096

**Published:** 2022-07-25

**Authors:** Tiemei Lu, Susanne Liese, Ludo Schoenmakers, Christoph A. Weber, Hiroaki Suzuki, Wilhelm T. S. Huck, Evan Spruijt

**Affiliations:** † Institute for Molecules and Materials, Radboud University, Heyendaalseweg 135, 6525 AJ, Nijmegen, The Netherlands; ‡ Institute of Physics, University of Augsburg, Universitätsstraße 1, 86159 Augsburg, Germany; § Department of Precision Mechanics, Faculty of Science and Engineering, Chuo University, Tokyo 112-8551, Japan

## Abstract

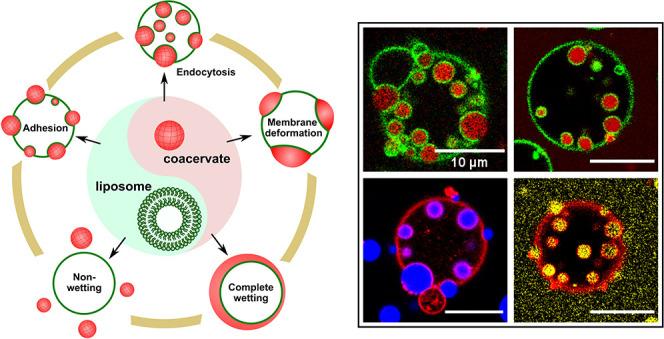

Recent studies have shown that the interactions between condensates and biological membranes are of functional importance. Here, we study how the interaction between complex coacervates and liposomes as model systems can lead to wetting, membrane deformation, and endocytosis. Depending on the interaction strength between coacervates and liposomes, the wetting behavior ranged from nonwetting to engulfment (endocytosis) and complete wetting. Endocytosis of coacervates was found to be a general phenomenon: coacervates made from a wide range of components could be taken up by liposomes. A simple theory taking into account surface energies and coacervate sizes can explain the observed morphologies. Our findings can help to better understand condensate–membrane interactions in cellular systems and provide new avenues for intracellular delivery using coacervates.

Membraneless organelles, such as nucleoli and stress granules, are condensates formed through liquid–liquid phase separation (LLPS)^1^ that play diverse roles in living cells. Although the absence of a lipid bilayer is a characteristic feature of these condensates (or coacervate droplets), recent studies have shown that droplet–membrane interactions have functional importance, for example, in T-cell receptor signal transduction,^2^ RNA granule transport,^3^ autophagy,^4^ the formation of protein storage vacuoles,^5^ or size control of ribonucleoprotein granules.^6^ It is thought that wetting is one of the key principles that governs the interaction between condensate droplets and membranes.^7,8^

Membranes and coacervates have also been combined in the field of artificial cells to create hierarchically organized compartments or hybrid protocells.^9^ Several groups have reported small coacervates encapsulated in liposomes without apparent wetting,^10^ but coacervate droplets can also partially wet^11^ and remodel membranes in such structures.^12^ In a different study, small phospholipid vesicles were found to assemble at the surface of large complex coacervate droplets without apparent deformation.^13^ However, when similar coacervate droplets were added to dried lipid films^14^ or mixed with ethanolic lipid solutions,^15^ membrane remodeling was observed, resulting in the assembly of a continuous, coacervate-supported phospholipid bilayer.^16^ In these examples, coacervate–membrane interactions and wetting play an important role in shaping the assembly of new structures.^17^ However, it remains unclear how droplet–membrane interactions could be used to direct membrane deformation and possibly induce endocytosis. Inspired by these recent findings, here we investigate the spatiotemporal organization of coacervate droplets and liposomes as a result of wetting.

To be able to tune the interactions between coacervates and liposomes in a continuous way, we used liposomes of 1-palmitoyl-2-oleoyl-*sn*-glycero-3-phosphocholine (POPC) with 10 wt % cholesterol and 0.17 wt % fluorescently labeled 1,2-Dioleoyl-sn-glycero-3-phosphoethanolamine (DOPE), containing varying fractions of positively and negatively charged lipids (1,2-dioleoyl-3-trimethylammoniumpropane (DOTAP) and 1-palmitoyl-2-oleoyl-*sn*-glycero-3-phospho-(1′-*rac*-glycerol) (POPG), respectively, Table S1), and complex coacervates with varying charge ratios (Table S2). By gradually increasing the membrane charge or coacervate composition, the droplet–liposome interaction strength can be changed from repulsive to strongly attractive (Table S3). We added a dispersion of small, polydisperse coacervate droplets (0.5–10 μm) to a sample of liposomes (5–50 μm) prepared by emulsion transfer inside a 30 μL microchannel (Figures S1 and S2) and observed the mixture by confocal fluorescence microscopy. We first investigated spermine/polyU coacervates mixed with positively charged, DOTAP-containing liposomes (Figure 1a), as these have previously been reported to interact.^13^ Interestingly, we observed that within an hour after mixing many liposomes had engulfed one or multiple coacervate droplets (Figure 1b). Intensity profiles (Figure 1c) and Z-stacks (Figure S5, Movies S1 and S2) demonstrate that the engulfed coacervates were fully coated with a lipid bilayer, indicative of endocytosis. Additional evidence for the complete encapsulation is shown in Figure 1d (Movie S3), where we dissolved all coacervates outside of the liposomes by adding salt.

**Figure 1:**
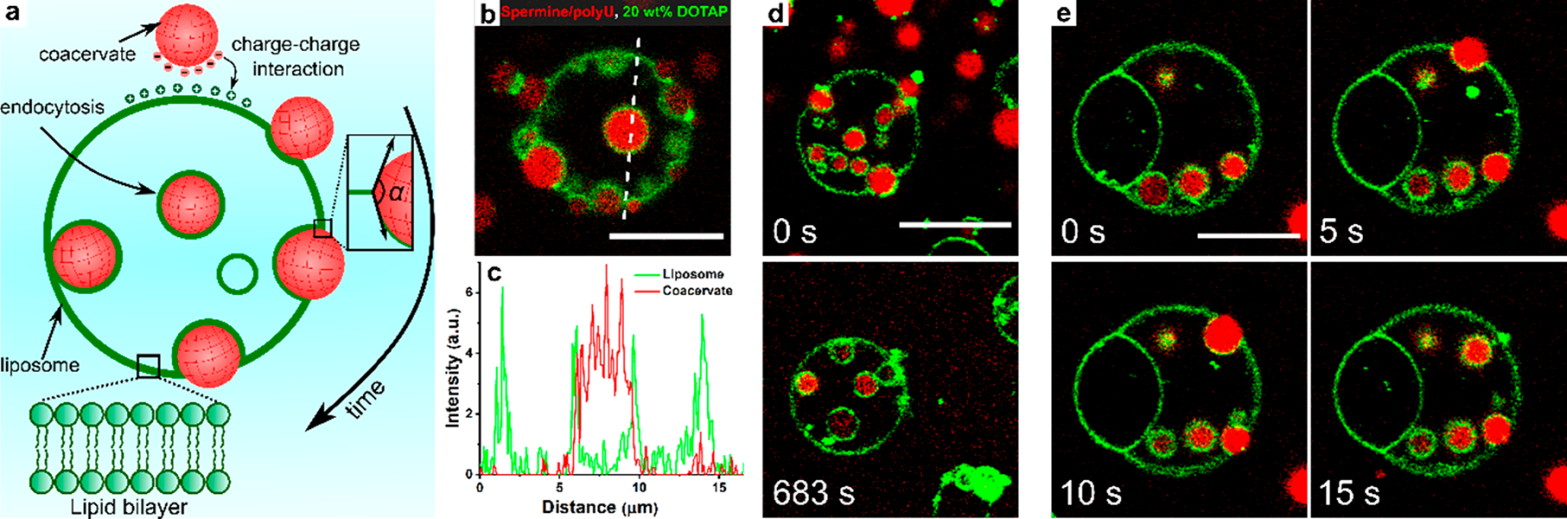
(a) Schematic illustration of endocytosis of coacervates by liposomes. (b) Spermine/polyU coacervates end up inside POPC_0.7_/cholesterol_0.1_/DOTAP_0.2_ liposomes after endocytosis. (c) Intensity profile along the dotted line in (b). (d) Snapshots of the system in (b) before and after addition of 1 μL of 3 M NaCl solution. (e) Time-lapse microscopy of endocytosis for the system in (b) (full images in Figures S3 and S4). Scale bars represent 10 μm.

To gain insight into the engulfment process, we followed the formation of lipid-coated coacervate “endosomes” using time-lapse microscopy. The engulfment is fast and proceeds via an apparent wetting transition (Figure 1e, Movie S4). As the coacervate contacts the liposome (5 s), the droplet (ca. 3.6 μm) partially wets the bilayer and adopts a transient lens-shaped form, characteristic of liquid droplets on liquid or soft interfaces.^18^ Within seconds, the lipid bilayer envelops the coacervate, like in endocytosis, resulting in complete engulfment after 15 s. This process is repeated for new coacervates, and after 20 min, tens of coacervates were engulfed (Figure S4c).

Endocytosis occurs for a range of coacervate and liposome sizes (observed for 0.9–7.7 and 7–22 μm, respectively, Movies S4–S6), but the wrapping time varies, taking up to 20 min in one case, possibly caused by multiple coacervates interacting with the liposome simultaneously (Figure S6b). The endocytosis of coacervates we observed bears remarkable similarity to recent work by Spustova et al, who found that local changes to membrane–surface interactions can lead to invaginations that grow into encapsulated subcompartments.^19^ Here, the coacervate droplets act as an adhesive surface for the membrane and as a template for the subcompartment.

To understand how the interaction strength affects the spatial organization of coacervates and liposomes, we systematically varied the membrane and coacervate composition. By increasing the fraction of positively charged DOTAP lipids from 0 to 35 wt %, we increased the interaction strength with the negatively charged coacervates (Figure S7) and found that the coacervates could cover the full range of possible wetting states on liposomes (Figure 2a–e). Without DOTAP (0 wt %), coacervates and liposomes do not interact (nonwetting). As we increased the DOTAP fraction, we first observed weak adhesion (10 wt %), followed by complete engulfment (20 wt %), spreading of coacervates into thin lenses that deform the membrane (30 wt %), and ultimately, complete wetting (35 wt %). We note that the transitions suggested by Figure 2a–e are gradual: both partially wetting coacervates and endosomes were found for DOTAP fractions between 10 and 20 wt % (Figure S10), as there is a distribution of the surface charge of both coacervates and liposomes (Table S3). Nevertheless, these results suggest that the droplet–membrane interaction strength, mediated by opposite charges in our model systems, is the key factor that governs the final geometry of interacting of condensates and liposomes.

**Figure 2:**
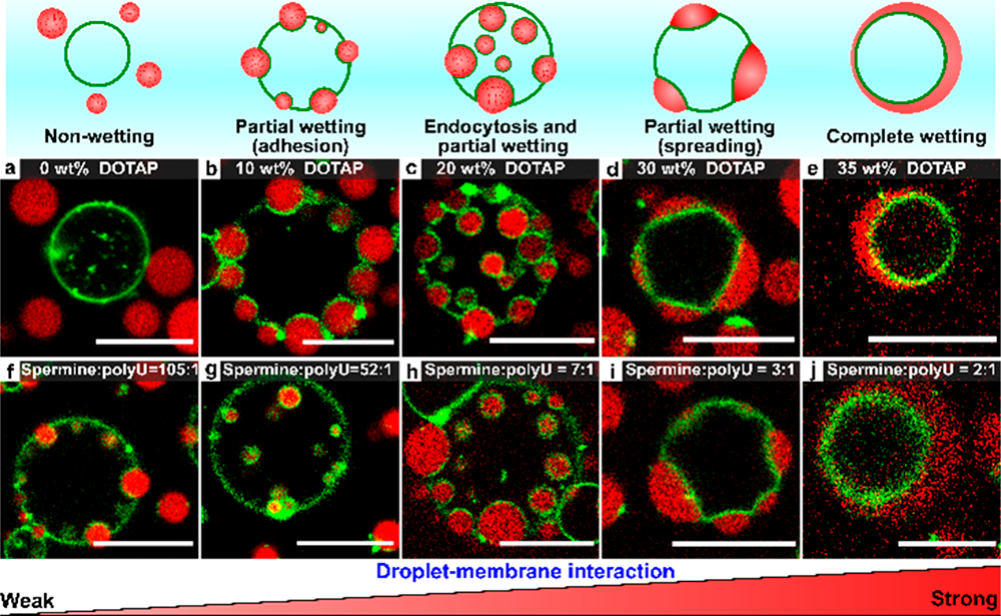
(a–e) Interaction of POPC/DOTAP liposomes and spermine/polyU coacervates for different DOTAP fractions. (f–j) Same as (a)–(e) for different spermine/polyU ratios interacting with 20 wt % DOTAP liposomes (full images in Figure S10). Scale bars represent 10 μm.

We also varied the droplet–membrane interaction by changing the spermine/polyU coacervate composition, and thereby the surface charge (Figure S7b). When these coacervates interacted with 20 wt % DOTAP liposomes, we observed the same wetting states as in the experiments with varying liposome compositions (Figure 2f–j), except for nonwetting, since coacervates with a net positive surface charge could not be formed. The size of the coacervate droplets appears to affect their engulfment by liposomes: at a 7:1 spermine/polyU ratio, the largest coacervates in our sample were found to partially wet the liposomes, while smaller coacervates were engulfed (Figure S10c,h). This suggests that the size ratio of droplets to liposomes may be another important factor governing the spatial organization of condensates and membranes.^20^

According to Figure 2, spatial organization of droplets on membranes can be tuned by the interaction strength between droplets and the membrane, regardless of the molecular details. To show that the different wetting states in Figure 2, and in particular endocytosis, are not limited to spermine/polyU coacervates, we varied the identity of both the liposome and coacervates. In all cases, we first tested the surface charge and critical salt concentration (CSC) (Figures S7 and S8, Table S3) to ensure we mixed droplets with liposomes that have an opposite and significant charge.

Figure 3a,b show that when polyU was replaced with another oligonucleotide (polyC or polyA), endocytosis was still possible. Interestingly, spermine/polyA coacervates were engulfed more easily by liposomes than spermine/polyC, despite their similar ζ-potential, probably because they are less “soft” (higher CSC).^21,22^ When spermine was replaced by oligoarginine (R_10_), the coacervate surface charge turned positive, and they could be engulfed by negatively charged liposomes containing POPG (Figure 3c). For another type of positively charged coacervates composed of poly(diallyldimethylammonium chloride)/ATP, we also observed all possible wetting states including endocytosis (Figures 3d, S12a–e). Finally, endocytosis and partial wetting were also observed for droplets made of disordered proteins (GFP-K_72_) and torula yeast RNA (Figure 3e, S12f–j) and two polymers with a high charge density (poly(allylamine hydrochloride) and poly-d-glutamate) (Figure 3f). In control experiments with coacervates that have the same surface charge as the liposomes, neither endocytosis nor partial wetting was observed (Figure S13), demonstrating that an attractive droplet–membrane interaction is required. It is clear that different complex coacervates can be engulfed by oppositely charged liposomes via endocytosis. These results motivated us to search for a theoretical underpinning that qualitatively explains endocytosis and other wetting states.

**Figure 3:**
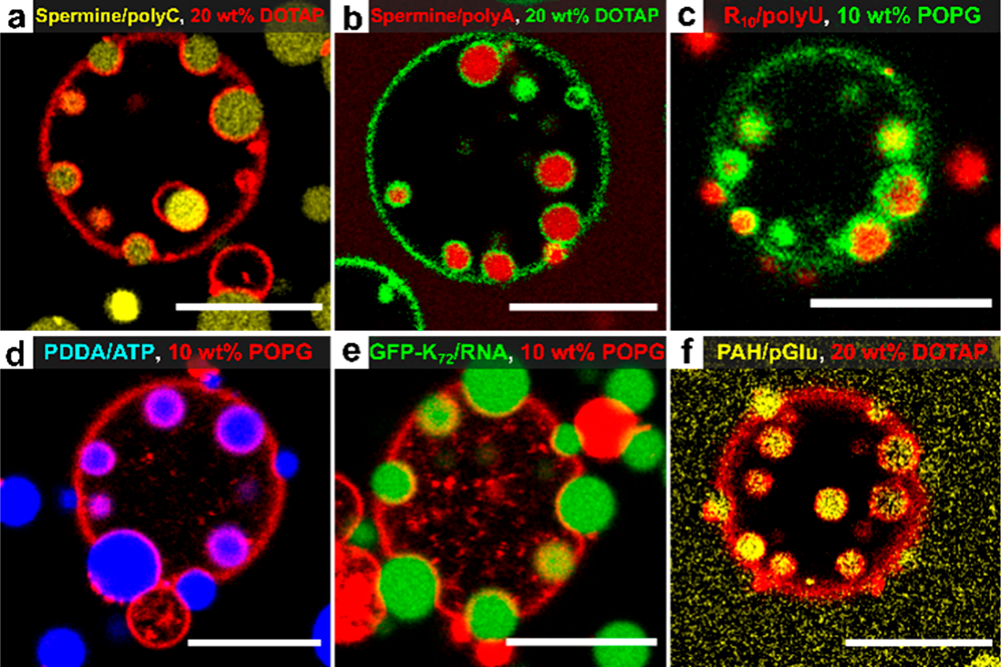
Composite images of different types of coacervates mixed with positively (a, b, f) or negatively (c–e) charged liposomes showing partial engulfment or complete endocytosis. Fluorescent labels and composition are indicated by the labels (full sample details and images in Table S2, Figure S11). Scale bars represent 10 μm.

Several theoretical works^22−24^ have addressed the interplay between wetting and membrane deformation. Most notably, Kusumaatmaja et al. examined endocytosis and budding of liquid-like droplets.^23^ They have shown that each droplet shape is well described by a spherical cap if the membrane tension is large compared to the droplet surface tension. For large droplets, the impact of the bending rigidity can be neglected. Thus, in analogy to the triple line between three liquid phases, the droplet shape is determined by the balance of surface tensions. For very large liposomes (*R*_L_ ≫ *R*_0_, where *R*_0_ is the size of the spherical coacervate droplet), the angle α that a droplet forms with the liposome surface is given by Neumann’s law (Figure 4a):
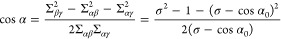
1where cos α_0_ = (Σ_βγ_ – Σ_αγ_)/Σ_αβ_ and the scaled membrane tension σ = Σ_βγ_/Σ_αβ_ are defined through the droplet and membrane surface tensions. The angles β and γ, used to quantify the membrane shape, are determined analogously.

**Figure 4:**
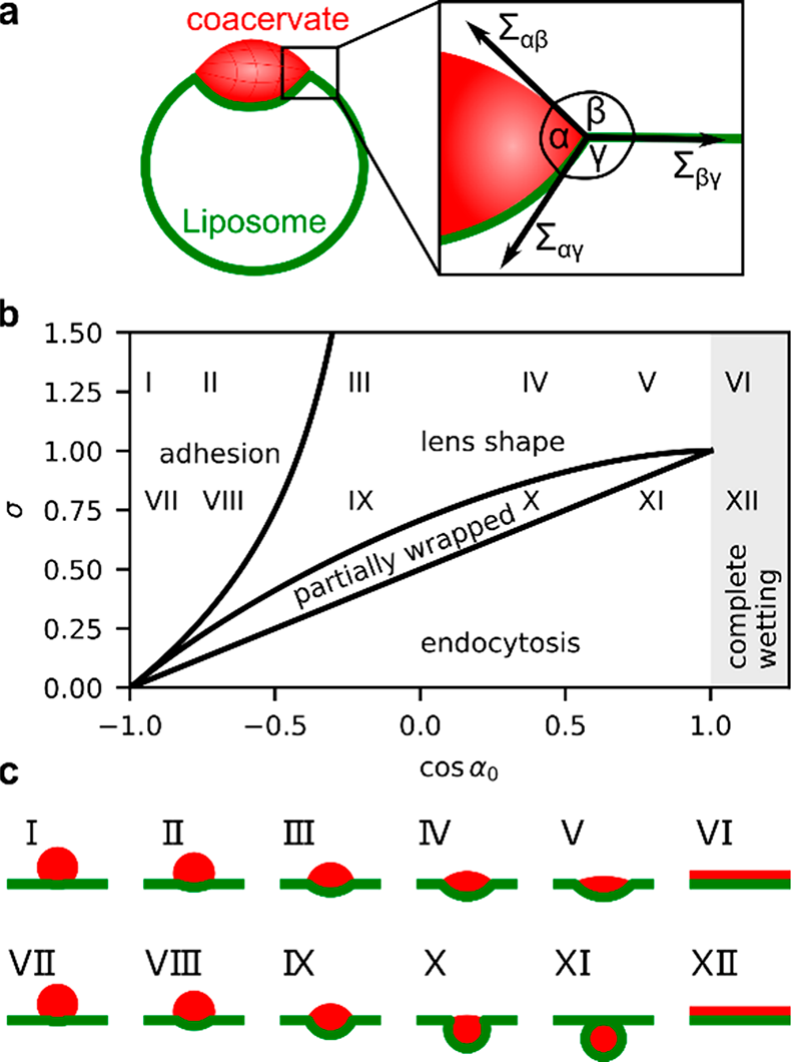
(a) Schematic of contact angles involved in wetting of a coacervate on a liposome. (b) The droplet shape diagram is determined by cos α_0_ and the scaled membrane tension σ. (c) Theoretical profiles of different shape types in (b) were calculated from eqs 1, S1, and S2.

This model predicts five different coacervate shapes, which we define depending on the angles α, β, and γ (Figure 4a): (1) endocytosis, where the droplet is completely engulfed by the membrane (γ → 0); (2) partially wrapped (γ < π/2); (3) lens shaped (α < π); (4) adhesion atop the liposome (β < π/2); and (5) complete wetting (β → 0). In Figure 4b, we show the coacervate shapes for different values of α_0_ and σ.

To understand how the molecular properties of the coacervates and liposomes studied here are linked to the shape parameters in Figure 4b, we take a closer look at their interpretation. Young’s contact angle α_0_ is proportional to the surface free energy of the contact region between droplet and membrane, which is related to the magnitude of the charge–charge interaction between the coacervates and liposomes. Taking spermine/polyU coacervates and DOTAP-containing liposomes as an example, going from left to right in Figure 4b thus corresponds to an increase in DOTAP fraction or, equivalently, an increase in polyU content, as confirmed by contact angle measurements on planar lipid bilayers (Figures S14 and S15). The measured coacervate contact angle α_0_ as a function of DOTAP fraction also allows us to estimate the characteristic value of the scaled membrane tension σ for which endocytosis occurs. For σ = 1.25, an increasing interaction strength (increasing cos α_0_) leads to a transition from spherical (shapes I and II) to lens-shaped droplets (shapes III*–*V) and complete wetting (shape VI). However, for a lower tension of σ = 0.75 (shapes VII–XII) we see a transition through all five shape types (Figure 4c).

Comparison with the experimentally observed shapes (Figures 2a–j and 3a–f) indicates that the membrane tension must be smaller than the droplet surface tension (σ < 1) for typical liposomes. Indeed, most complex coacervates have surface tensions (0.01–1 mN/m)^25^ that exceed typical membrane tensions of phospholipid bilayers (0.1–10 μN/m).^26^ However, for intracellular condensates much lower surface tensions have been reported,^25^ and cell membrane tensions may be higher, depending on cell type. Hence, the fate of condensates interacting with membranes *in vivo* will depend strongly on their exact composition: all shapes depicted in Figure 4, including endocytosis, are within reach of typical tension values reported in the literature.^27^

Droplet endocytosis reduces the volume of the liposome. Interestingly, for finite-size liposomes, the volume change causes a contribution to the energy that acts like a size-dependent membrane tension, where the effective membrane tension scales as σ → σ(1 + *R*_0_/(2*R*_L_)) (derivation in the SI). We hypothesize that this effect impedes endocytosis, as observed experimentally (Figures S10c, S11b). In qualitative terms, an initially large, strongly interacting liposome can take up multiple droplets, which leads to a decrease of its radius. The resulting increase in membrane tension may hinder endocytosis of additional droplets, although partitioning of charged lipids may also play a role.

In summary, we have demonstrated that coacervate droplets can wet and deform lipid membrane and be taken up via endocytosis, driven by an attractive droplet–membrane interaction. Endocytosis of coacervate droplets could be a powerful tool to deliver nutrients or genetic material into artificial cells or to create membrane-bound artificial organelles.

## Supporting Information Available

Methods and materials, supplementary tables, figures, and extended theory of coacervate–liposome interactions, and captions of movies (PDF)
Movie S1: Z-stack rotation along the *Y* axis (AVI)
Movie S2: Zoom-in of the white dashed square area in Figure S5g (AVI)
Movie S3: Dissolution of spermine/polyU coacervates outside of liposomes droplets (AVI)
Movie S4: Process of spermine/polyU coacervates going into positively charged POPC/DOTAP (20 wt %) liposomes by endocytosis (AVI)
Movie S5: The same composition as Movie S4, but the size of the coacervates and liposomes is different (AVI)
Movie S6: The same sample as Movie S5, but in a different position (AVI)

## Supplementary Material

ja2c04096_si_001.pdf

ja2c04096_si_002.avi

ja2c04096_si_003.avi

ja2c04096_si_004.avi

ja2c04096_si_005.avi

ja2c04096_si_006.avi

ja2c04096_si_007.avi
