# Supplementary material for: Endocytosis of Coacervates into Liposomes
Source: J Am Chem Soc. 2022 Jul 25;144(30):13451–5. doi: 10.1021/jacs.2c04096 (PMC9354246; doi:10.1021/jacs.2c04096)
Supplement: Supplementary file 1 — ja2c04096_si_001.pdf [file ja2c04096_si_001.pdf]

Supplementary information

Endocytosis of coacervates into liposomes

Tiemei Lu[1], Susanne Liese[2], Ludo Schoenmakers[1], Christoph A. Weber[2], Hiroaki Suzuki[3], Wilhelm T. S. Huck[1] and Evan Spruijt[1,*]

[1]Institute for Molecules and Materials, Radboud University, Heyendaalseweg 135, 6525 AJ, Nijmegen, The Netherlands

[2]Institute of Physics, University of Augsburg, Universitätsstr. 1, 86159 Augsburg, Germany

[3]Department of Precision Mechanics, Faculty of Science and Engineering, Chuo University, Tokyo 112-8551, Japan

Contents

1. Methods and Materials 2
a. Materials 2
b. polyU/polyA labelling 2
c. Stock solutions 3
d. Preparation of coacervates 3
e. Preparation of coacervates 4
f. Confocal microscopy experiments 4
g. Critical salt concentrations 4
h. Measuring the surface charge of coacervates and liposomes 5
i. Supported lipid bilayer formation 5
j. Contact angle measurement 6
2. Supplementary Tables 7
3. Supplementary Figures 9
a. Experimental setups 9
b. Partial and full wrapping of coacervates by liposomes leading to endosomes 10
c. Characterization of coacervate and liposomes charge, stability and material properties 13
d. Different wetting states at different coacervate and membrane composition 14
e. Coacervate-liposome interactions in different systems 15
f. Contact angle measurements of coacervates on planar lipid bilayers 17
4. Supplementary theory of coacervate-liposome interactions 18
a. Droplet shapes 18
b. Impact of the liposome size on the effective membrane tension 19
5. Supplementary movie captions 22
References 23

1. Methods and Materials

a. Materials

All materials were purchased from Sigma-Aldrich unless otherwise specified. For different types of coacervates, we used: spermine (TCI Europe N.V.), adenosine 5′-triphosphate disodium salt hydrate (ATP), polyadenylic acid potassium salt (polyA), polyuridylic acid potassium salt (polyU), polycytidylic acid potassium salt (polyC), ribonucleic acid (RNA, from torula yeast), poly-L-arginine trifluoroacetic acid (R_{10}, 2.7 kDa, CASLO), poly(diallyl dimethylammonium chloride) (PDDA, 200-350 kDa, 20 wt% solution in H_2O), poly(allylamine hydrochloride) (PAH, 58 kDa) and poly-D-glutamate (pGlu, 5.6 kDa, PDI 1.06, Alamanda Polymers) was described elsewhere.[1] All coacervates were prepared in solutions containing D-(+)-glucose monohydrate (Glucose), sodium chloride (NaCl), Tris(hydroxymethyl)-aminomethane hydrochloride (Tris-HCl) and magnesium chloride hexahydrate ($MgCl_2 \cdot 6H_2O$).

For visualisation purposes, pyranine, labelled polyG$_{11}$(G5-Super-dG-G5-Cy3Sp) was purchased from Integrated DNA Technologies (IDT), polyU and polyA were labelled with AlexaFluor647-hydrazide (polyU-Alexa 647, polyA-Alexa 647), tetramethylrhodamine labelled PAH (PAH-TAMRA) was prepared by carbodiimide mediated coupling reaction with EDC and NHS following a previous report.[2]

For the formation of liposomes, we used 1-palmitoyl-2-oleoyl-*sn*-glycero-3-phosphocholine (16:0-18:1 PC, POPC), 1-palmitoyl-2-oleoyl-*sn*-glycero-3-phospho-(1´-*rac*-glycerol) (sodium salt) (16:0-18:1 PG, POPG) and 1,2-dioleoyl-3-trimethylammonium-propane (chloride salt) (18:1 TAP, DOTAP), all purchased from Avanti polar lipid. ATTO-488 and ATTO-655 labelled 1,2-dioleoyl-*sn*-glycero-3-phosphoethanolamine (Atto-488-DOPE and Atto-655-DOPE) were obtained from ATTO-TEC. Chloroform ($CHCl_3$, Fisher Scientific, ≥99%), sucrose (bioultra, for molecular biology, ≥99.5%), cholesterol (≥99%) and liquid paraffin (Wako, Japan) were also used. The structure of different lipids can be found in Table S1.

For the modification of microscopy slides, we used poly(L-lysine)-*graft*-poly(ethylene glycol) (PLL-*g*-PEG, SuSoS AG), µ-slides with 18 wells and 6 channels (No. 1.5, polymer coverslip, Ibidi GmbH). For both two types of slides, the surface was cleaned using a plasma cleaner, then adding 30 µL/100 µL of a 0.1 mg/mL PLL-*g*-PEG solution, which dissolved in 10 mM 4-(2-hydroxyethyl)-1-piperazineethanesulfonic acid (HEPES) (pH = 7.4), to each well/channel, covering it with the lid and incubating slides at room temperature for 24 h. Finally, slides were washed with MQ water and dried with compressed N_2.

b. polyU/polyA labelling

The polyU and polyA were labelled with AlexaFluor647-hydrazide using a periodate oxidation reaction. Take polyU for an example, 72.5 µL of polyU (20 mg/mL), 14.5 µL of nuclease free water, 3.33 µL of 3 M sodium acetate (pH = 5.2), and 10 µL of 25 mM sodium periodate (in nuclease free water, freshly prepared on the day) were added into a 1.5 mL microcentrifuge tube (1.5 mL, Eppendorf). The mixture was incubated on ice for 50 min. Subsequently, the polyU was spun down (14,000 g at 4 ºC for 10 min, twice) by using

Amicon spin concentrators (30 kDa). After that, the concentrated polyU was mixed with 25 nmol AlexaFluor647-hydrazide dye and 100 mM sodium acetate (pH = 5.2). The mixture was incubated at 4 ºC for at least 6h/overnight in the dark, after which the labelled polyU was purified with several rounds of sample concentration using an Amicon spin concentrator (30 kDa) and diluting with nuclease free water during the concentration process. The purity was checked by 1% agarose gel electrophoresis and the concentration was calculated by using the Nanodrop. The concentration of labelled polyU and polyA were 21.2 mg/mL and 4.0 mg/mL, respectively.

c. Stock solutions

All of the following chemicals were dissolved in Milli-Q water (MQ, 18.2 MΩ cm): ATP (50 mM), spermine (100 mM), R_{10} (10 mg/mL, 36.76 mM in monomer), PDDA (50 mg/mL, 0.31 M in monomer units), PAH (10 mg/mL, 0.11 M in monomer units), pGlu (13 mg/mL, 0.10 M in monomer units), pyranine (1 mM), PAH-TAMRA (10 mM), Tris-HCl (0.5 M, pH = 7.4), $MgCl_2$ (50 mM), NaCl (3 M), sucrose (1 M), glucose (1 M). The labelled DNA oligonucleotide $polyG_{11}$ was dissolved in nuclease-free water at a concentration of 100 μM. In addition, the following stock solutions were also prepared in nuclease-free water: polyA (6.4 mg/mL), polyC (6.4 mg/mL), polyU (10 mg/mL), torula yeast RNA (3.4 mg/mL), GFP-K_{72} (140 μM). Lipid stock solutions in $CHCl_3$ were prepared by drying the purchased lipid solution in the flask using a rotary evaporator and then redissolving the dried lipid in the desired amount of $CHCl_3$ to a final concentration of 50 mg/mL for POPG and POPC and 25 mg/mL for DOTAP. ATTO-488-DOPE, ATTO-655-DOPE and cholesterol powders were dissolved in $CHCl_3$ at a concentration of 1 mg/mL, 1 mg/mL and 60 mg/mL, respectively. All the solutions were stored at −20 °C, except Tris-HCl, $MgCl_2$, NaCl, glucose and sucrose, which were stored at 4 °C. All lipid solutions were transferred to HPLC vials and kept under argon.

d. Preparation of coacervates

The liposomes were prepared based on the water-in-oil (W/O) emulsion transfer method, as has been reported previously.[3] Briefly, POPC, POPG and cholesterol, or POPC, DOTAP and cholesterol in chloroform stocks were dissolved in 400 μL liquid paraffin at a weight ratio of 8:1:1 (1.5 mg/mL total lipid concentration) or 7:2:1 (3.0 mg/mL total lipid concentration), respectively. The weight fraction of POPG changed from 0 to 0.5, and DOTAP changed from 0 to 0.35. For observing under a microscope, 0.17 wt% ATTO-dye labeled phospholipids (ATTO-488-DOPE or ATTO-655-DOPE) was included in the lipid mixture. After adding 20 μL of so-called inner solution, which contained 300 mM sucrose, 50 mM Tris-HCl (pH = 7.4), 5 mM $MgCl_2$ and NaCl (the concentration of NaCl is corresponding to the coacervate droplets), the mixture was vortexed for 40 s to obtain a W/O emulsion. Then, the emulsion was gently placed on top of 400 μL of the so-called outer solution, which contained 300 mM glucose and the same concentration of Tris-HCl (pH = 7.4), $MgCl_2$ and NaCl as the inner solution in a new microcentrifuge tube for 5 min. Subsequently, this two-layered solution was centrifuged at 10,000 rpm (9,391 ×g) for 10 min at

20 ºC to obtain liposomes precipitated at the bottom of microcentrifuge tube as a pellet. Around 100 to 200 μL of the precipitated liposomes suspension was collected through a hole pierced using a needle at the bottom of the microcentrifuge tube. The obtained liposomes suspension was further centrifuged at 5,000 rpm (2,348 ×g) at 20 °C for 5 min. The supernatant was removed from the microcentrifuge tube as far as possible whilst not damaging the newly formed pellet, which was then redispersed in 20-100 μL of the outer solution. Finally, dispersed liposomes with 5-50 μm in diameter were obtained, as shown in Figure S1.

e. Preparation of coacervates

Typically, coacervates were prepared by first mixing NaCl, Tris-HCl, $MgCl_2$, MQ, glucose and the desired type of negatively charged polyU, polyC, polyA, RNA, ATP or pGlu in a microcentrifuge tube (0.5 mL, Eppendorf) at the required concentration, followed by the addition of positively charged spermine, R_{10}, GFP-K_{72}, PDDA and PAH from their respective stock solutions. The total volume of the mixtures was 20 μL. The final concentration of polyU, polyC, polyA, RNA, ATP and pGlu in the mixture are 0.25-3.0 mg/mL, 1.0 mg/mL, 1.0 mg/mL, 0.30 mg/mL, 14.8 mM and 2.2 mM, respectively. The final concentration of spermine, R_{10}, GFP-K_{72}, PDDA and PAH in the mixture are 5-20 mM, 3.7 mM, 10 μM, 4.9 mM and 2.2 mM, respectively. The final concentration of NaCl is 0 or 50 mM and the final concentration of Tris-HCl, $MgCl_2$ and glucose are 50 mM, 5 mM and 300 mM, respectively. Mixing was done by gentle pipetting (3×).

f. Confocal microscopy experiments

Samples of interaction between coacervates and liposomes for the microscopy experiments were prepared in microcentrifuge tubes. Normally, 20 μL of a freshly prepared coacervate or liposome dispersion was added directly to a modified μ-slide chamber for checking the formation and concentration. Then, the liposomes were diluted with the outer solution to a suitable concentration and the diluted liposomes were added to the μ-slide channel (6×). The slide was placed onto the microscope and the coacervate droplets were then added from one side of the channel.

Images and videos were obtained by using a Leica TCS Sp8X confocal microscope, equipped with HyDs and PMTs detectors and a pulsed white light laser. HC PL APO CS2 40×/1.10 (water), 63×/1.40 (oil) and 100×/1.40 (oil) objectives were used. Samples were visualized by an excitation of 405 nm (pyranine), 501 nm (ATTO-488), 554nm (cy3), 573nm (RhoB) and 649 nm (ATTO-655, Alexa-647, cy5).

g. Critical salt concentrations

The critical salt concentration of single-phase coacervates was measured on a microplate reader (Tecan Spark), equipped with a microinjector, as described elsewhere.[4] Briefly, turbidity of a coacervate solution with a total starting volume of 100 μL (C_{NaCl} = 0 M) was monitored as a function of the concentration of NaCl at a wavelength of 600 nm and a temperature of 25 ± 0.5 °C in 96-well plates (Greiner Bio-one, clear flat-bottom wells) by titration with NaCl (0.3-3.0 M) in 2 or 5 μL steps. Samples were incubated for 10 min

at test temperature. After each injection step, the samples were mixed by shaking for 3 s, followed by equilibration for 5 s, and shaken for another 3 s before every readout. The critical point was calculated by extrapolating the first-order derivative at the inflection point to zero turbidity. Note that this critical salt concentration does not take into account ions from other sources than the added NaCl, and the actual critical ionic strength may be slightly higher.

h. Measuring the surface charge of coacervates and liposomes

Zeta potential measurements were conducted on a Malvern DLS-Zetasizer. Coacervates were formed in similar buffer conditions to those for the interaction with liposomes experiments (50 mM Tris-HCl at pH = 7.4, 5 mM $MgCl_2$, 300 mM glucose, and/or 50 mM NaCl). The positively charged and negatively charged molecules for forming the coacervates were each diluted 2 to 10 times. After coacervates were formed, samples were injected into a disposable folded capillary cell (DTS1070) and measured at 25 °C. Three measurements were taken, each consisting of 100 runs. For the measurements of liposomes, they were diluted 3 to 8 times after formation and the other test methods and conditions were the same as for the coacervates.

i. Supported lipid bilayer formation

Supported lipid bilayers (SLBs) were prepared following the vesicle fusion method described in Kurniawan et al.[5] Briefly, lipids (POPC, DOTAP and cholesterol) dissolved in chloroform were mixed in the same ratio as Figure 2a-d, in which the concentration of DOTAP is 0-50 wt%, in a small round bottom flask and dried under a nitrogen stream. The dried lipids were further dried in a vacuum dryer for 4 hours. The dried lipid film was dissolved into SLB buffer (50 mM Tris-HCl at pH = 7.4, 5 mM $MgCl_2$, 300 mM glucose and 50 mM NaCl) at 2 mg/mL using a vortex. The lipid mixture was sonicated using a probe sonicator (MSE Soniprep 150) at 4 µm amplitude, 15 s on/off, on ice for 10 cycles. Right before SLB formation, the vesicles were diluted to 0.5 mg/mL in SLB buffer and extruded through a 100 nm polycarbonate filter (Avestin) to form monodisperse small unilamellar vesicles (SUVs). To form the SLB, a mica disk (Nano-Tec) was glued to a glass coverslip. Several layers of mica were peeled off using scotch tape. The clean mica surface was activated using a plasma cleaner (Diener Electronics Femto) at full power and time for 2 cycles. A small PDMS ring was placed around the mica disk onto the glass coverslip and the mica was incubated with 400 µL of 0.5 mg/mL lipids for 20 min at room temperature. After incubation, the remaining SUVs were washed away by carefully adding and removing 500 µl of SLB buffer for a total of eight times, taking care to always keep the SLB in buffer. Presence of lipids at the mica surface was tested using confocal microscopy (Leica SP8 Liachroic), as shown in Figure S14.

j. Contact angle measurement

For the contact angle measurements, spermine/polyU (13:1) coacervate droplets (condensed phase) were deposited on SLBs with various DOTAP compositions (0-50 wt%). The contact angle was determined using an FTA1000 Drop Shape Instrument (First Ten Angstroms). Briefly, spermine/polyU (13:1) coacervate droplets were prepared in 5 mL microcentrifuge tubes (5 mL, Eppendorf) and incubated at 30 °C for 5 min for droplet formation. Subsequently, the coacervate droplets were spun down in a centrifuge (Eppendorf 5810R) at 4,000 rpm (841 ×g), 10 min, at 30 °C. The top dilute phase was carefully removed and the bottom condensed phase was stored at 30 °C until use. Using a blow dryer, the metal stage on the drop shape instrument that holds the sample was preheated to around 28-30 °C. The previously prepared SLB was placed into a preheated custom-made glass box and preheated SLB buffer was added to completely cover the slide. The PDMS ring was removed carefully and the box was placed on the metal stage. Using a plastic spatula and a fine point needle, a droplet of condensed phase was placed on top of the submerged SLB. Images were taken at 0, 5 and 10 min, respectively. The temperature of the buffer was monitored using a temperature probe. The temperature was kept around 28 °C by heating the stage as needed. Contact angles were determined using Image J (with contact angle plugins),[6] contact angle = 180 – average Theta (average of left and right Theta). As the droplets need time to equilibrate, we analyzed the images at 10 min, which as shown in Figure S15.

2. Supplementary Tables

Table S1. Structures of the lipids used in this study.

Lipids	Structure
POPC (16:0-18:1)	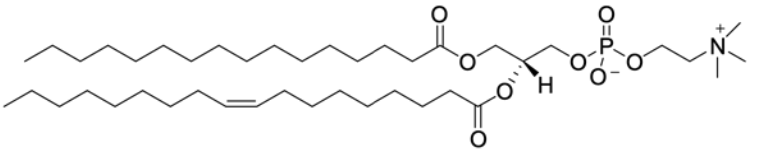
POPG (16:0-18:1)	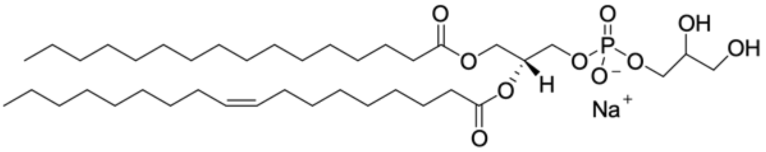
DOTAP (18:1 TAP)	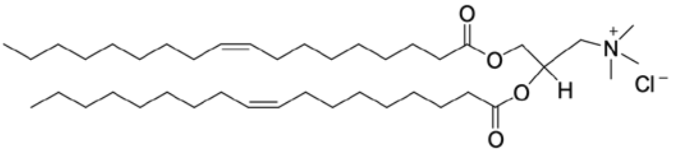
Cholesterol	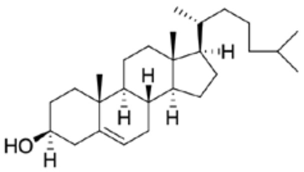

Table S2. The coacervates and liposomes combinations used in this study. (All liposomes contain 10 wt% cholesterol.)

Types of coacervates	Positively charged liposomes	Negatively charged liposomes
Spermine/polyU (red, polyU-Alexa 647)	POPC/DOTAP (0-35 wt%) (green = ATTO-488-DOPE)	POPC/POPG (50 wt%) (green = ATTO-488-DOPE)
Spermine/polyC (yellow = polyG$_{11}$-Cy3)	POPC/DOTAP (20 wt%) (red = ATTO-655-DOPE)	-
Spermine/polyA (red = polyA-Alexa 647)	POPC/DOTAP (20 wt%) (green = ATTO-488-DOPE)	-
R$_{10}$/polyU (red, polyU-Alexa 647)	-	POPC/POPG (10 wt%) (green = ATTO-488-DOPE)
PDDA/ATP (blue = free pyranine dye labelled coacervates)	-	POPC/POPG (5-50 wt%) (red = ATTO-655-DOPE)
GFP-K$_{72}$/RNA	POPC/DOTAP (10 wt%) (red = ATTO-655-DOPE)	POPC/POPG (5-50 wt%) (red = ATTO-655-DOPE)
PAH/pGlu (yellow= PAH-TAMRA)	POPC/DOTAP (20 wt%) (red = ATTO-655-DOPE)	-

Table S3. Zeta potential of different combinations of liposomes, zeta potential and critical salt concentrations (CSC) of different types of coacervate droplets. Turbidity measurements and zeta potential were performed in triplicate and error represents the standard deviation (n = 3).

Liposome combination	Weight fraction of DOTAP/POPG	ζ - potential (mV)
POPC/DOTAP	0 to 0.35	1.8 to 28.9 (Figure S7a)
POPC/POPG	0 to 0.5	3.3 to – 23.2 (Figure S8)
**Coacervate combination**	**ζ - potential (mV)**	**Critical salt concentration (mM)**
Spermine/polyU	− 5.7 to − 15.7 (Figure S7b)	160 to 290 (Figure S7c)
Spermine/polyC	− 6.26 ± 3.06	110.0 ± 2.9
Spermine/polyA	− 8.04 ± 0.55	505.7 ± 6.9
PAH/pGlu	− 24.53 ± 1.89	2784.9 ± 13.6
GFP-K_{72}/torula yeast RNA	8.02 ±2.45	98.9 ± 2.5
R_{10}/polyU	9.69 ± 0.88	1103.9 ± 8.1
PDDA/ATP	18.23 ± 3.55	22.6 ± 1.6

3. Supplementary Figures

a. Experimental setups

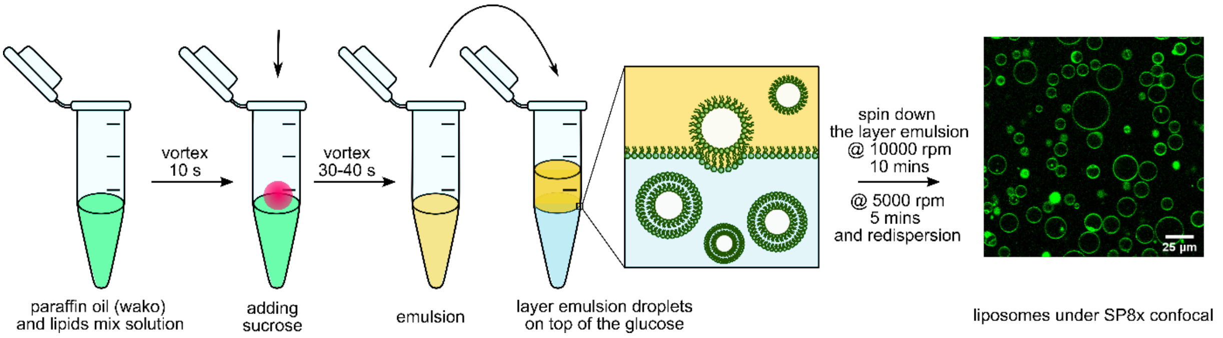

Figure S1: Schematic illustration of liposomes preparation and confocal fluorescence microscopy of POPC/DOTAP (20 wt%) liposomes with fluorescence from ATTO-488-DOPE.

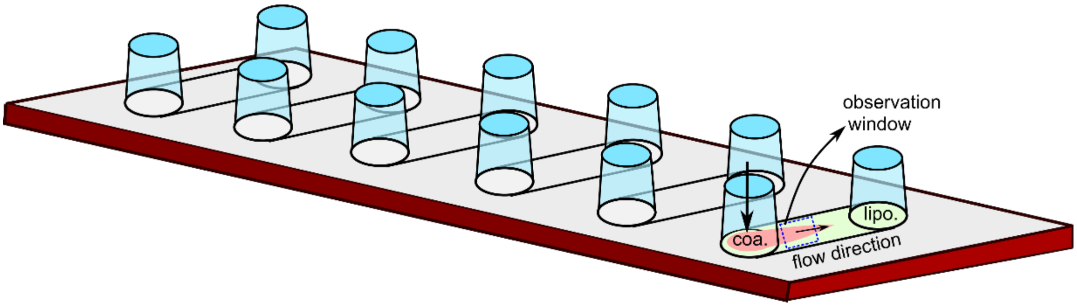

Figure S2: 20 µL liposomes (lipo.) are added to the channel first and the channel is fixed to the confocal microscope before 20 µL coacervates (coa.) are added. The blue dashed box is the window where we observe our samples.

b. Partial and full wrapping of coacervates by liposomes leading to endosomes

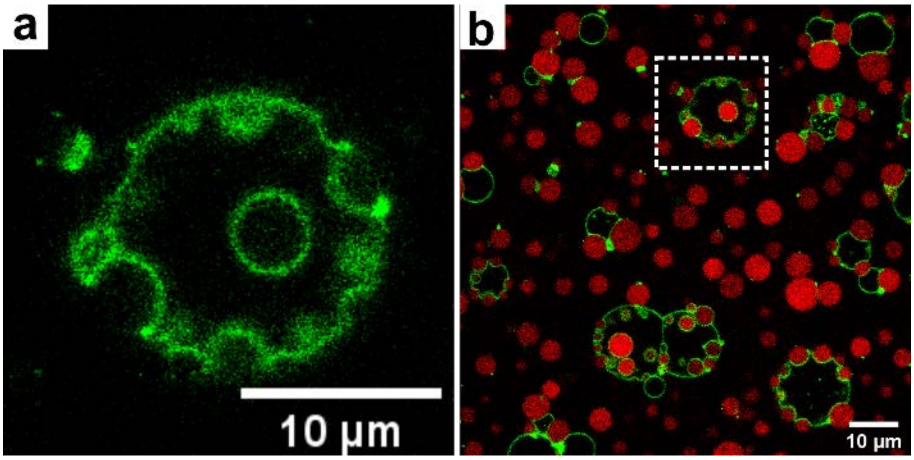

Figure S3: (a) The green channel of liposome in Figure 1b. (b) The original full image of Figure 1b, which is a zoom in from the white dashed square area in b.

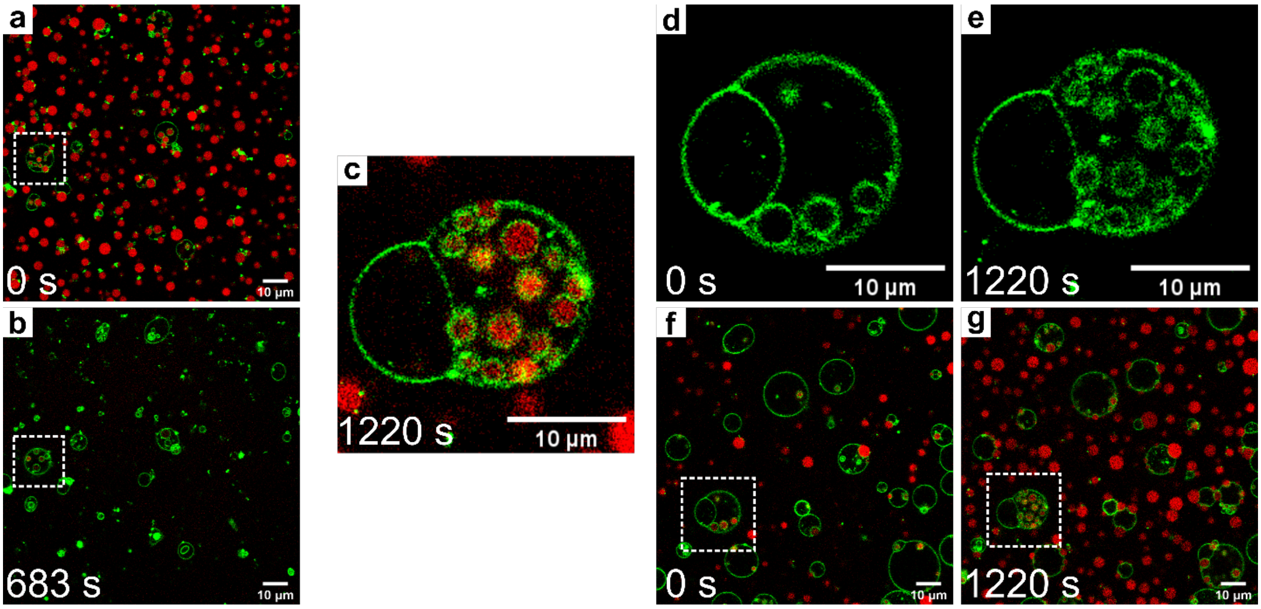

Figure S4: (a) the original full image of Figure 1d at t = 0s, (b) the original full image of Figure 1d at t = 683s, (c) The final stage of the endocytosis process in Figure 1e. (d)The green channel of liposome in Figure 1e at t = 0s, (e) the green channel of liposome in c, (f) the original full image of Figure 1e at t = 0s and (g) the original full image of c. Figure 1d and 1e are a zoom in from the white dashed square areas.

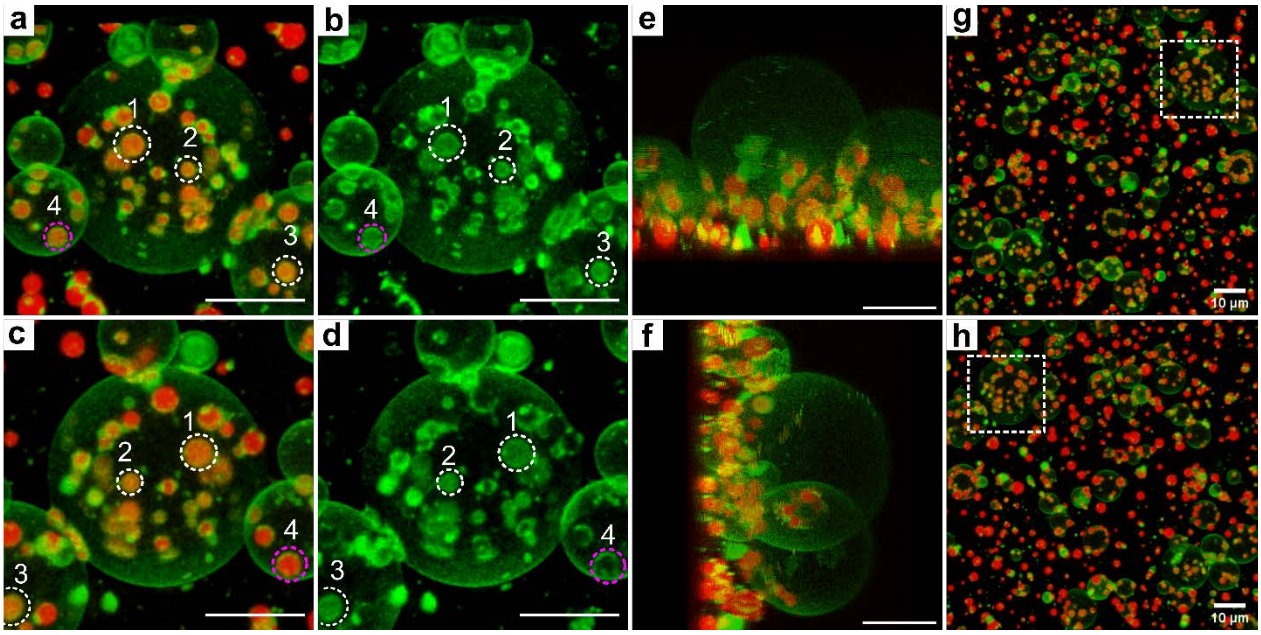

Figure S5: 3D images of spermine/polyU coacervates encapsulated by POPC/DOTAP (20 wt%) liposomes are from Z-stack. (a) A zoom in from the white dashed square area in (g), (b) the green channel of liposome in (a), (c) a zoom in from the white dashed square area in (h), (d) the green channel of liposome in (c), (e) z-x 3D image and (f) z-y 3D image from the white dashed square area in (g), (g) the original full image of (a), (h) obtained after flipping figure (g) by 180°. Scale bars represent 10 µm in (a-f).

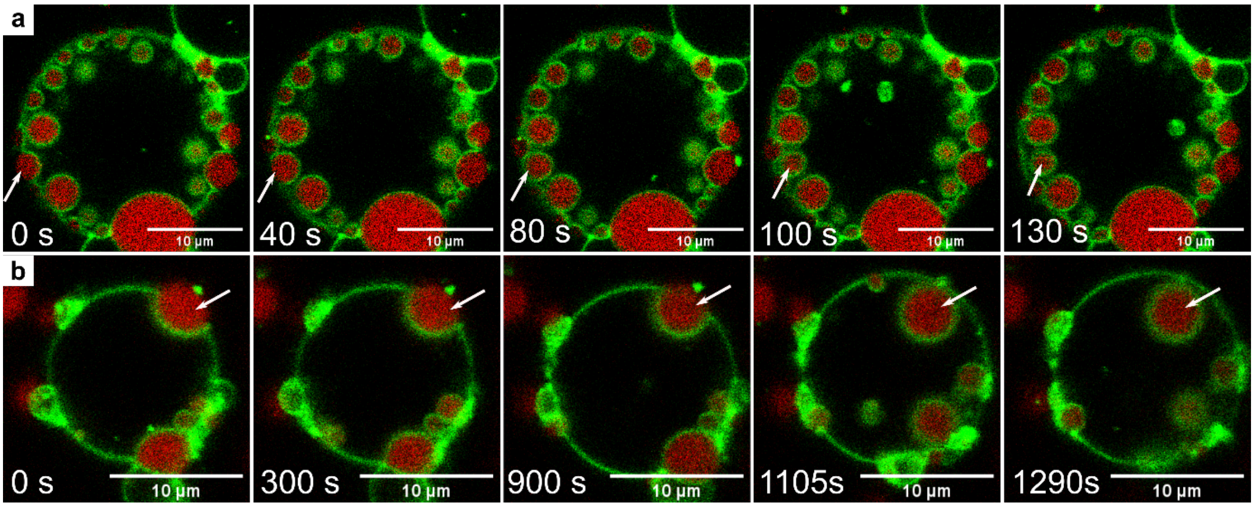

Figure S6: Snapshots of the process of spermine/polyU coacervate encapsulated by POPC/DOTAP (20 wt%) liposome. The size of coacervate droplet (indicated by the arrow) and liposome is (a) 3.30 µm and 21.79 µm, respectively; (b) 4.18 µm and 14.17 µm, respectively.

Note to Figures S4-S6

We note that the wrapping times of coacervates can vary significantly. While wrapping was complete in 15 seconds in Figure 1e, in one other case, it took nearly 20 minutes. It appears that the coacervates that are wrapped slowly spend a substantial time in a partially wrapped state (Figure S6b). Possibly, the strength of the interaction between the coacervate droplet and the membrane is weaker than in the example shown in

Figure 1e. This could occur in the same sample, because not all coacervates and liposomes have the same surface charge: Figure S7 and Table S3 indicate that the zeta potentials of both coacervates and liposomes have a distribution with a 10-50% standard deviation. Indeed, we also observed coacervates that remained in a partially wetted state in the sample containing 20% DOTAP, likely because their interaction was not strong enough to ensure full wrapping. Finally, it is also possible that the number of coacervates interacting with the same liposome simultaneously has an effect on the wrapping time: adhering coacervates could lead to an increase of the effective membrane tension and slow down wrapping.

We quantified the changes of the membrane area upon endocytosis, and found that it remains to a good approximation constant during the engulfment of a single coacervate droplet: relative perimeter changes in Figure 1e, S6a and S6b: -1%, +2%, +3% (±8%), respectively. However, when multiple coacervates (about 20) had entered the liposome by endocytosis, the membrane area was significantly smaller, as can be seen in Figure S4d-e (final perimeter change: -12 ± 8%). We do note that these images are 2D cross sections of a 3D liposome. Slight shifting of the focal plane could also lead to changes in the apparent perimeter or area.

c. Characterization of coacervate and liposomes charge, stability and material properties

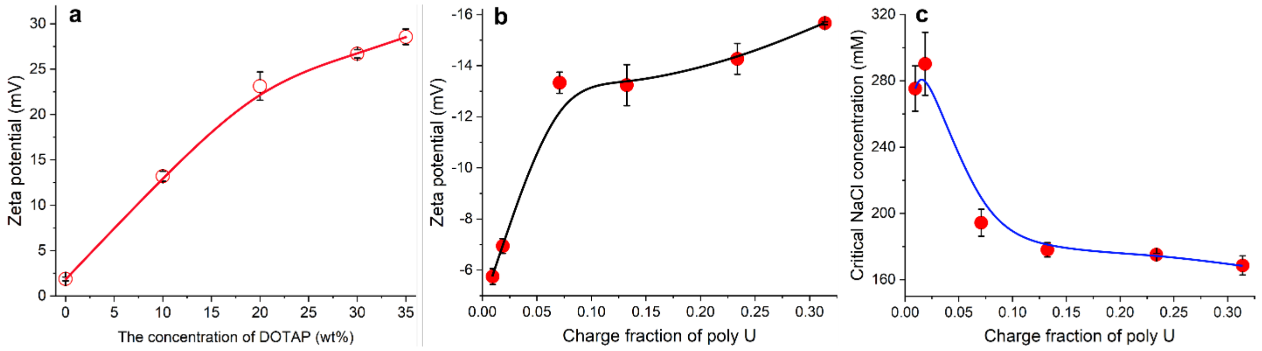

Figure S7: (a) Zeta-potential of different concentration of DOTAP liposomes. (b) Zeta-potential of different charge fraction of polyU in spermine/polyU coacervates. (c) Critical salt concentrations of different charge fraction of polyU in spermine/polyU coacervates, which were determined by turbidity measurements. And lines are drawn as guide to the eye.

Note to Figure S7

Changing the spermine/polyU ratio not only changes the surface charge, but possibly also the material properties of the coacervates. We therefore determined their critical salt concentration (CSC, Figure S7c), as an indirect measure of the expected changes in the interfacial tension, viscosity and density of the coacervates. The CSC decreases with increasing polyU content, suggesting that coacervates with a higher polyU content have a lower interfacial tension, viscosity and density, and may therefore be ‘softer’. As softer droplets are more prone to spreading and require stronger adhesion energy to achieve successful internalization, the formation of endosomes may happen for slightly different interaction strengths and droplet sizes for other condensates.

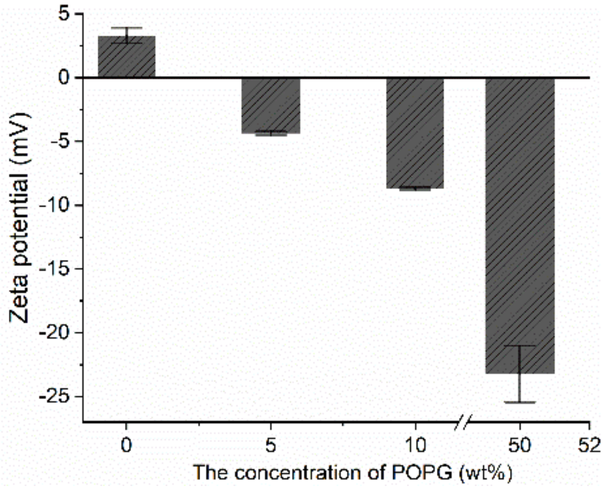

Figure S8: Zeta-potential of POPG-containing liposomes.

d. Different wetting states at different coacervate and membrane composition

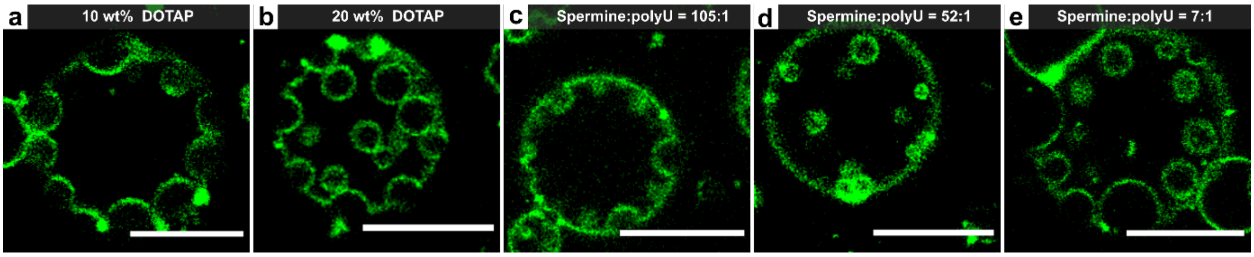

Figure S9: Images of liposome channel with droplets partially encapsulate by lipids or forming an endocytosis structure. (cf. Fig. 2b,c,f,g,h). All scale bars represent 10 μm.

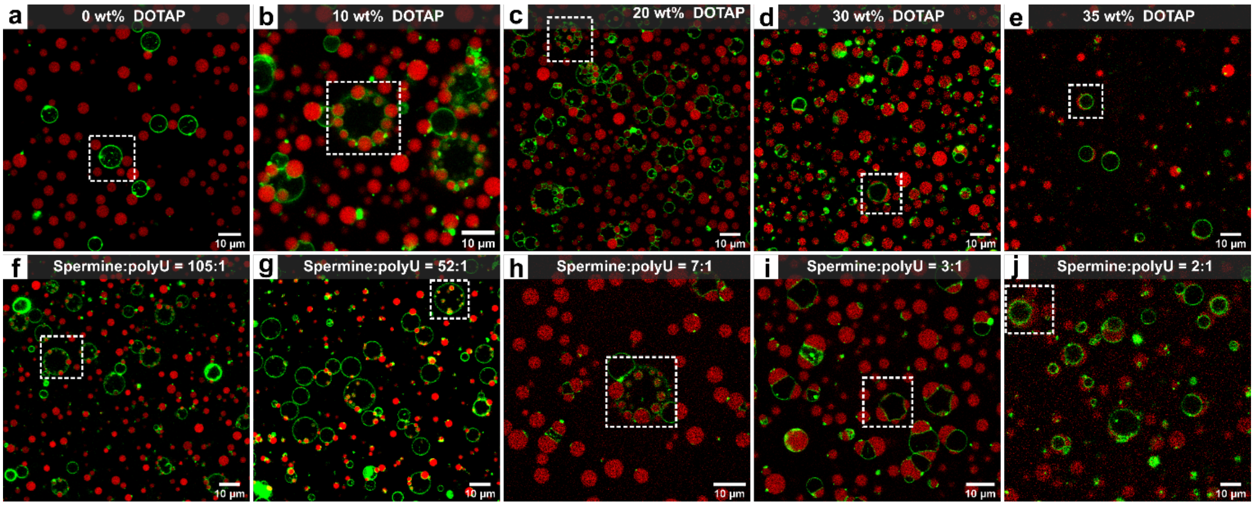

Figure S10: The original full images of Figure 2, which are a zoom in from the white dashed square areas.

Note to Figure S10

We found that endocytosis and other wetting phenomena can occur for a wide range of liposome sizes (diameters between 7 and 22 μm) and coacervate sizes (diameters between 0.9 and 7.7 μm). To provide more insight into the suggested size effect of endocytosis, we analyzed the sizes of the coacervate droplets that displayed endocytosis and partial wetting in all samples with liposomes containing 20% DOTAP and coacervates composed of spermine/polyU 13:1 and 7:1 (for example shown in Figure S10c,h). We found that endocytosis occurs for coacervates that are on average slightly smaller, while partial wetting occurs for larger coacervates (see histogram below) However, there is substantial overlap between both distributions and the difference between the averages is less than a standard deviation. This is likely the result of the distribution of surface charge of both coacervates and liposomes (Figure S7-8, Table S3), which implies that the strength of the interaction is not identical for all coacervates and liposomes in a single sample.

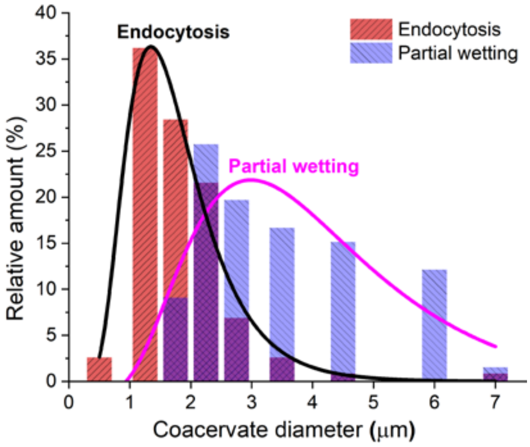

e. Coacervate-liposome interactions in different systems

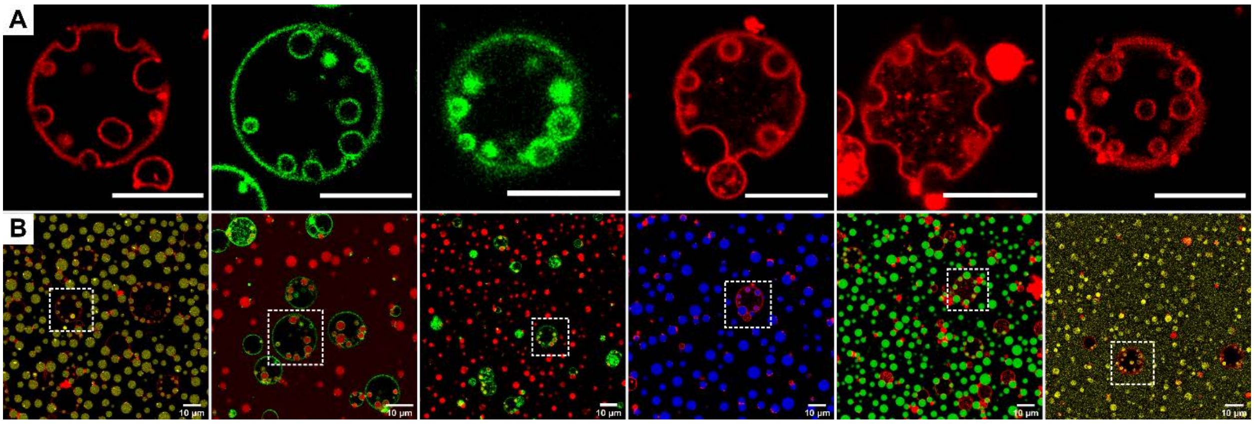

Figure S11: (A) Images of liposomes channel of Figure 3. All scale bars represent 10 µm. (B) The original full images of Figure 3, which are a zoom in from the white dashed square areas.

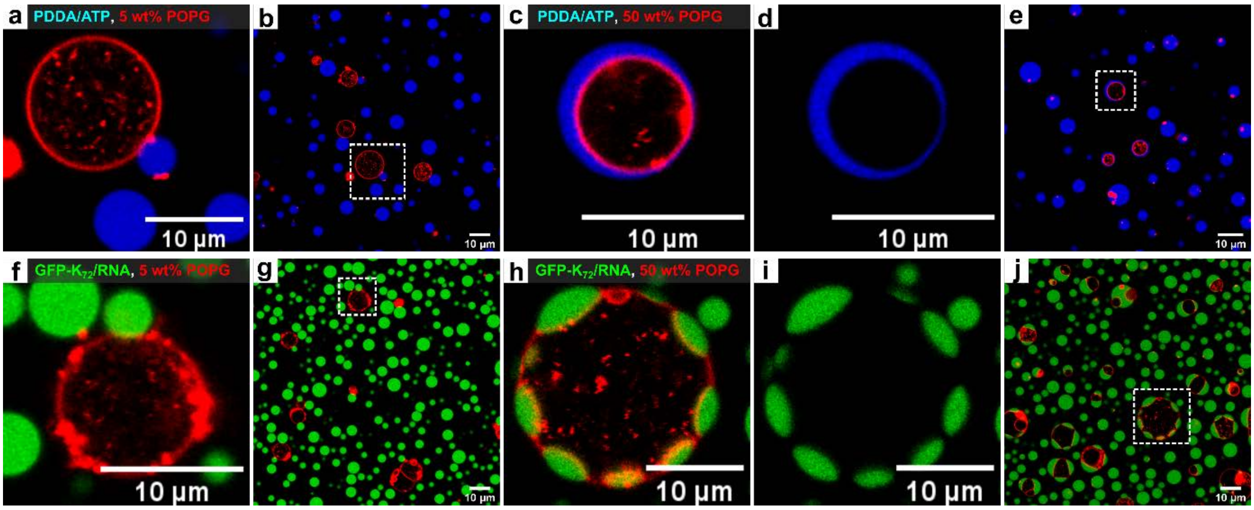

Figure S12: Images show PDDA/ATP and GFP-K_{72}/RNA interacts with different concentration of POPG liposomes, respectively. (a) PDDA/ATP (blue) with POPC/POPG (5 wt%) liposomes (red), no interactions between them. (b) The original full images of (a), which is a zoom in from the white dashed square area. (c) PDDA/ATP (blue) with POPC/POPG (50 wt%) liposomes (red), coacervate droplets completely wetting the liposome. (d) coacervate channel show that droplets fully coat the lipid membrane, internalize a vesicle-like structure. (e) The original full images of (c), which is a zoom in from the white dashed square area. (f) GFP-K_{72}/RNA (green) with POPC/POPG (5 wt%) liposomes (red), there is no interaction between them. (g) The original full images of (f), which is a zoom in from the white dashed square area. (h) GFP-K_{72}/RNA (green) with POPC/POPG (50 wt%) liposomes (red), droplets partial wetting on the surface of liposomes membrane can be observed. (i) coacervate channel shows that droplets are partially wetting around the liposome. (j) The original full images of (h), which is a zoom in from the white dashed square area.

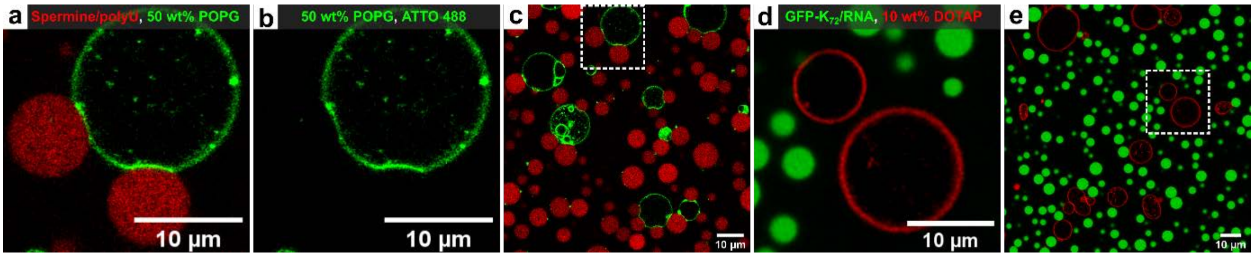

Figure S13: Control experiments of the interaction between coacervate droplets with like-charged liposomes. (a) Negatively charged coacervates spermine/polyU(red) with negatively charged POPC/POPG (50 wt%) liposomes (green). (b) The green channel of liposome in (a), only a small deformation of the liposome can be observed. (c) The original full images of (a), which is a zoom in from the white dashed square area. (d) Positively charged coacervates GFP-K_{72}/RNA with positively charged POPC/DOTAP (20 wt%) liposomes, they exclude each other. (e) The original full images of (d), which is a zoom in from the white dashed square area.

Note to Figures S11-S13

Figure 3a,b shows that when polyU was replaced with another oligonucleotide (polyC or polyA), endocytosis was still possible. Interestingly, spermine/polyA coacervates were easier to be engulfed by liposomes than spermine/polyC, despite their similar ζ-potential, probably because of their less soft character (higher CSC). When spermine was replaced by oligoarginine (R_{10}), the surface charge of the coacervates turned positive, and they could be engulfed by negatively charged liposomes containing POPG (Figure 3c). When we inverted the relative sizes of the coacervate components by replacing the small molecule spermine by a polymer, PDDA, and the polyU by a small molecule, ATP, and mixed these PDDA/ATP coacervates with POPG-containing liposomes, we observed the full range of wetting states including endocytosis (Figure 3d, Figure S12a-e). Endocytosis and partial wetting were also observed for droplets made of disordered proteins (GFP-K_{72}) and torula yeast RNA (Figure 3e, Figure S12f-j). However, contrary to PDDA/ATP coacervates, complete wetting could not be achieved with GFP-K_{72}/RNA by varying the POPG content, as the ζ-potential of PDDA/ATP is 127% higher than GFP-K_{72}/RNA. Finally, when we replaced both coacervate components by polymers with a high charge density (PAH and pGlu), endocytosis was still possible (Figure 3f). In two control experiments with coacervates that have the same surface charge as the liposomes, neither endocytosis nor partial wetting was observed (Figure S13), demonstrating that an attractive interaction between droplets and membranes is required.

f. Contact angle measurements of coacervates on planar lipid bilayers

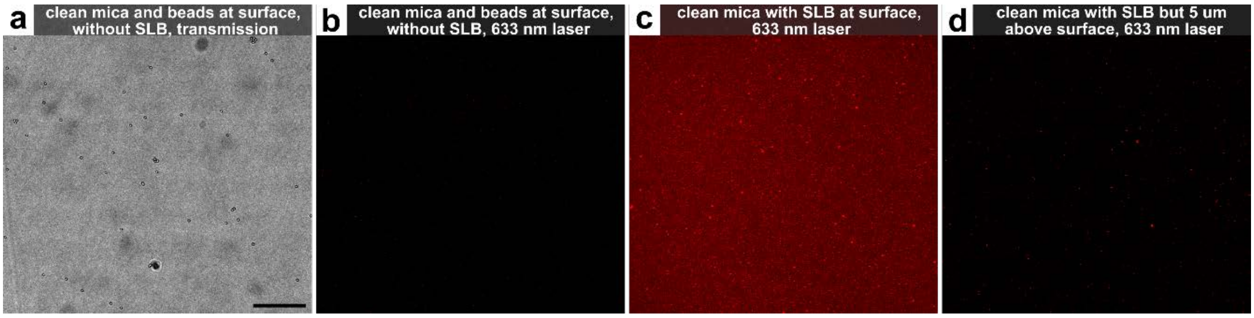

Figure S14: Sp8 confocal (a) bright-field image of beads at clean mica surface without SLB, (b) the same sample and same position as (a) but showing no fluorescence at 633 nm, (c) SLB labelled with ATTO-655-DOPE on a clean mica surface, without beads, but strong fluorescence proving the presence of SLB on the mica surface, (d) the same sample as (c), but moving the stage 5 μm above the objective, there is almost no fluorescence.

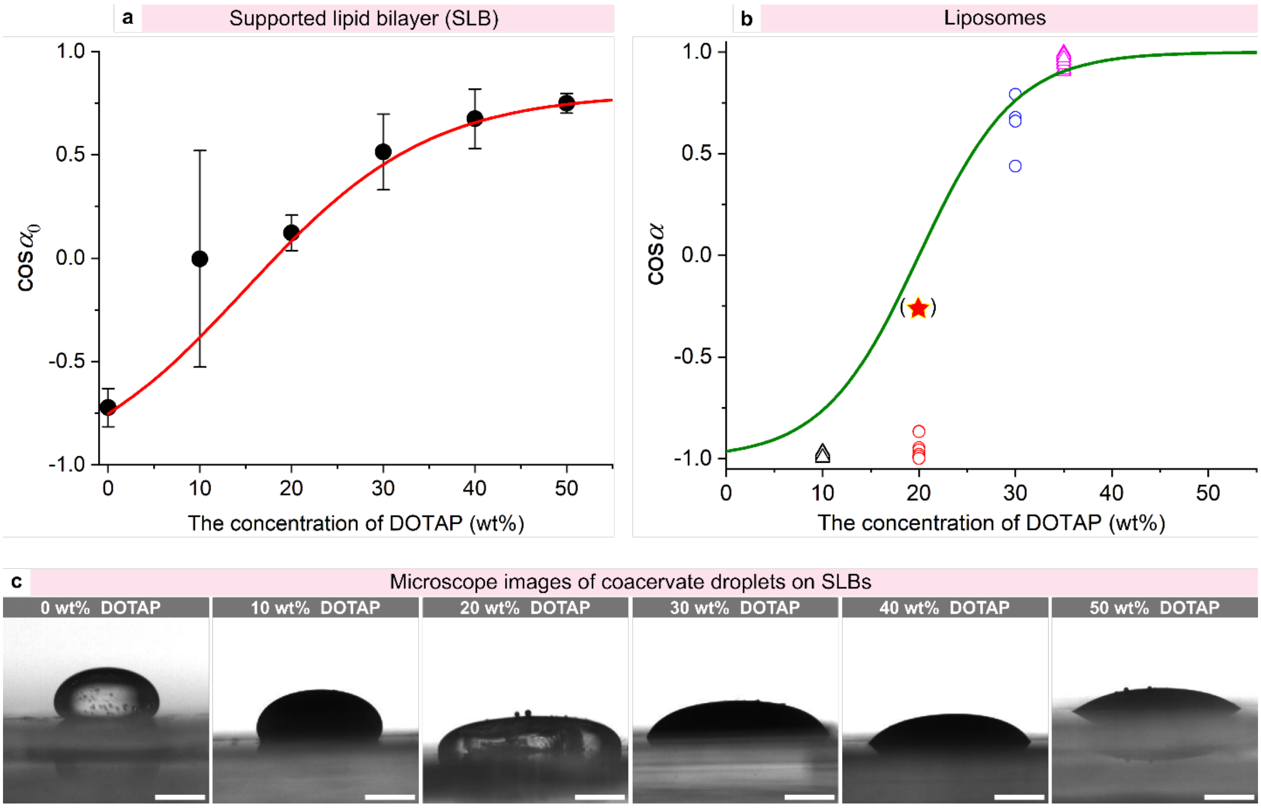

Figure S15: (A) A plot showing the variation in cos α_0 between spermine/polyU (13:1) coacervates and SLBs as a result of changes in DOTAP concentration. (Based on contact angle measurements of droplets that have been equilibrated for 10 min.) (B) A scatter plot showing the variation in cos α between spermine/polyU (13:1) coacervates and liposomes as a result of changes in DOTAP concentration. Scatter data is extracted from microscopy images as shown for instance in Figure 2b-e. Symbols △ represents 10 wt% DOTAP (26 data points). Symbols ○ represents 20 wt% DOTAP (19 data points). The asterisk ★ indicates that coacervates showing endocytosis were not included in the data, as α could not be determined for these droplets. Therefore, the values of α shown by the open red circles are an underestimation of the actual interfacial energy (cos α). Symbols ○ represents 30 wt% DOTAP (4 data points). Symbols △ represents 35 wt% DOTAP (44 data points). Solid lines are logistic functions drawn here as guide to the eye. (c) Typical sideview images of spermine/polyU (13:1) coacervate droplets on SLBs at 10 min, scalebars represent 1 mm.

4. Supplementary theory of coacervate-liposome interactions

a. Droplet shapes

One of the crucial findings of Kusumaatmaja et al. is that the shape of a large droplet wrapped by an elastic membrane is determined in analogy to the shape of a droplet at the interface of two other liquids.[7] This result is based on the rational that the bending energy depends only marginally on the droplet radius, while the area of the droplet and the contact area increase quadratically with the droplet radius. The balance of the related surface tensions thus dominates the droplet shape. The angle α that the droplet forms at the contact line is then given by Neumann's law:

$$\cos\alpha = \frac{\Sigma_{\beta\gamma}^2 - \Sigma_{\alpha\beta}^2 - \Sigma_{\alpha\gamma}^2}{2\Sigma_{\alpha\beta}\Sigma_{\alpha\gamma}} = \frac{\sigma^2 - 1 - (\sigma - \cos\alpha_0)^2}{2(\sigma - \cos\alpha_0)}, \tag{S1}$$

$$\cos\beta = \frac{\Sigma_{\alpha\gamma}^2 - \Sigma_{\alpha\beta}^2 - \Sigma_{\beta\gamma}^2}{2\Sigma_{\alpha\beta}\Sigma_{\beta\gamma}} = \frac{(\sigma - \cos\alpha_0)^2 - \sigma^2 - 1}{2\sigma} \tag{S2}$$

$$\cos\gamma = \frac{\Sigma_{\alpha\beta}^2 - \Sigma_{\alpha\gamma}^2 - \Sigma_{\beta\gamma}^2}{2\Sigma_{\alpha\gamma}\Sigma_{\beta\gamma}} = \frac{1 - (\sigma - \cos\alpha_0)^2 - \sigma^2}{2\sigma(\sigma - \cos\alpha_0)} \tag{S3}$$

We distinguish five shape types. The parameter regimes in which the different shapes are found are as follows.

Complete wetting:

$$\cos\alpha_0 > 1 \tag{S4}$$

Endocytosis, is found for $\beta > \pi$. Based on Eq. S2 we find:

$$\sigma < \frac{1 + \cos\alpha_0}{2} \quad \text{and} \quad \cos\alpha_0 < 1 \tag{S5}$$

Partially wrapped shapes are defined by $\gamma < \pi/2$. Based on Eq. S3 we find:

$$\sigma < \frac{\cos\alpha_0}{2} + \sqrt{\frac{1}{2} - \frac{\cos^2\alpha_0}{4}} \quad \text{and} \quad \cos\alpha_0 < 1 \tag{S6}$$

The scenario of a droplet shapes with $\beta < \pi/2$ are denoted adhesion. Based on Eq. S2 we find:

$$\sigma > \frac{\cos^2\alpha_0 - 1}{2\cos\alpha_0} \quad \text{and} \quad \cos\alpha_0 < 0 \tag{S7}$$

For all other parameters we find lens-shaped droplets.

b. Impact of the liposome size on the effective membrane tension

To derive the impact of the liposome size on the effective membrane tension, we first consider the limiting case of an infinite, planar membrane. Following the argument by Kusumaatmaja et al.,[7] the bending energy is neglected for sufficiently large droplets. The membrane energy thus only contains surface tension terms. To describe the shape of the droplet and the membrane, we use the spherical cap approximation. The energy difference, ΔE, between a droplet that wets and deforms the membrane and a spherical coacervate droplet adjacent to a planar membrane reads:

$$\Delta E = 2\pi R_\beta^2(1+\cos\beta)\Sigma_{\alpha\beta} + 2\pi R_\gamma^2(1+\cos\gamma)\Sigma_{\alpha\gamma} - 4\pi R_0^2\Sigma_{\alpha\beta} - \pi R_\gamma^2 \sin^2\gamma\,\Sigma_{\beta\gamma} \qquad \text{(S8)}$$

with $\Sigma_{\alpha\beta}$, $\Sigma_{\beta\gamma}$ and $\Sigma_{\alpha\gamma}$ the surface and membrane tensions as depicted on Figure 4a in the main text. R_0 (or R_{coac}) denotes the radius of the spherical droplet. The radii R_β, R_γ and the angles β, γ are schematically depicted in Figure S16a, Eq. S8 is equivalently written as:

$$\frac{\Delta E}{4\pi R_0^2\Sigma_{\alpha\beta}} = \left(\frac{R_\beta}{R_0}\right)^2\frac{1+\cos\beta}{2} - \cos\alpha_0\left(\frac{R_\gamma}{R_0}\right)^2\frac{1+\cos\gamma}{2} + \sigma\left(\frac{R_\gamma}{R_0}\right)^2\left(\frac{1+\cos\gamma}{2}\right)^2 \qquad \text{(S9)}$$

with $\cos\alpha_0 = (\Sigma_{\beta\gamma} - \Sigma_{\alpha\gamma})/\Sigma_{\alpha\beta}$ and $\sigma = \Sigma_{\beta\gamma}/\Sigma_{\alpha\beta}$.

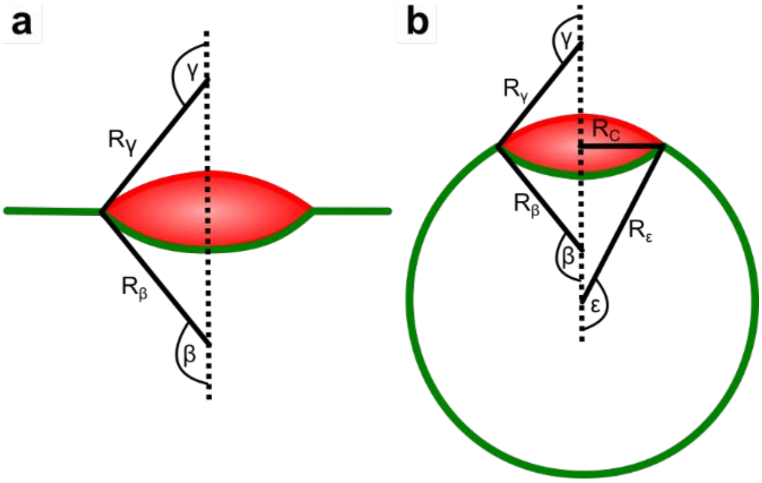

Figure S16: (a) A droplet that wets and deforms an infinite, initially planar membrane is depicted schematically. (b) A droplet that wets and deforms an initially spherical liposome is depicted schematically.

In the second step, we consider a finite liposome with an initially spherical shape with radius R_L (or R_{lipo}). in Figure S16b shows schematically a droplet that wets and deforms a liposome with the radii R_β, R_γ, R_ϵ and the angles β, γ, ϵ. The radius of the contact line is denoted as R_C. The geometry of the system implies the following relations:

$$R_C^2 = R_\beta^2(1-\cos^2\beta) = R_\gamma^2(1-\cos^2\gamma) = R_\epsilon^2(1-\cos^2\epsilon) \qquad \text{(S10)}$$

The droplet surface tension is denoted as $\tilde{\Sigma}_{\alpha\beta}$, the membrane tensions in the contact region and in the droplet-free region are denoted as $\tilde{\Sigma}_{\alpha\gamma}$ and $\tilde{\Sigma}_{\beta\gamma}$. P is the pressure across the lipid membrane. The energy difference between a droplet in contact with a liposome and a spherical droplet adjacent to a spherical liposome reads:

$$\begin{aligned} \Delta E = {} & 2\pi\Sigma_{\alpha\beta}R_\beta(1+\cos\beta) + 2\pi\tilde{\Sigma}_{\alpha\gamma}R_\gamma^2(1+\cos\gamma) + 2\pi\tilde{\Sigma}_{\beta\gamma}R_\epsilon^2(1-\cos\epsilon) - PV \\ & - 4\pi\tilde{\Sigma}_{\alpha\beta}R_0^2 - 4\pi\tilde{\Sigma}_{\beta\gamma}R_L^2 + \frac{4}{3}\pi R_L^3 P \end{aligned} \tag{S11}$$

with

$$V = \frac{4}{3}\pi R_\epsilon^3 - \frac{\pi}{3}R_\epsilon^3(2-\cos\epsilon)(1+\cos\epsilon)^2 - \frac{\pi}{3}R_\gamma^3(2-\cos\gamma)(1+\cos\gamma)^2 \tag{S12}$$

The energy of a spherical liposome, E_L, is give by

$$E_L = 4\pi\tilde{\Sigma}_{\beta\gamma}R_L^2 - \frac{3}{4}\pi R_L^3 P \tag{S13}$$

Minimizing with respect to R_L leads to the Laplace equation, which allows to replace the pressure in Eq. S11 by:

$$P = \frac{2\tilde{\Sigma}_{\beta\gamma}}{R_L} \tag{S14}$$

Lipid membranes are known to exhibits only a small area extensibility. We therefore take the liposome area as fixed, which implies:

$$R_\epsilon^2\left(\frac{1-\cos\epsilon}{2}\right) = R_L^2 - R_\gamma^2\left(\frac{1+\cos\gamma}{2}\right) \tag{S15}$$

The volume of the liposome, Eq. S12, can be rewritten as:

$$\begin{aligned} V &= \frac{1}{R_\epsilon}\left[\frac{4}{3}\pi R_\epsilon^4 - \frac{\pi}{3}R_\epsilon^4(2-\cos\epsilon)(1+\cos\epsilon)^2 - \frac{\pi}{3}R_\epsilon R_\gamma^3(2-\cos\gamma)(1+\cos\gamma)^2\right] \\ &= \frac{1}{R_\epsilon}\left[\frac{4}{3}\pi R_\epsilon^4\left(\frac{(1-\cos\epsilon)^2}{4} + \left(\frac{1-\cos\epsilon}{2}\right)\left(\frac{1-\cos^2\epsilon}{2}\right)\right) \right. \\ &\quad \left. - \frac{\pi}{3}R_\epsilon R_\gamma^3(2-\cos\gamma)(1+\cos\gamma)^2\right] \end{aligned} \tag{S16}$$

Inserting Eqs. S10 and S15 leads to

$$\begin{aligned} VR_\epsilon = {} & \frac{4}{3}\pi\left(R_L^2 - R_\gamma^2\frac{1+\cos\gamma}{2}\right)^2 + \frac{4}{3}\pi\left(R_L^2 - R_\gamma^2\frac{1+\cos\gamma}{2}\right)R_\gamma^2\left(\frac{1-\cos^2\gamma}{2}\right) \\ & - \frac{\pi}{3}R_\epsilon R_\gamma^3(2-\cos\gamma)(1+\cos\gamma)^2 \end{aligned} \tag{S17}$$

Inserting Eqs. S14, S15 and S17 into S11 leads to

$$\frac{\Delta E}{4\pi R_0^2 \Sigma_{\alpha\beta}} = \left(\frac{R_\beta}{R_0}\right)^2 \frac{1+\cos\beta}{2} - \left(\frac{\tilde{\Sigma}_{\beta\gamma} - \tilde{\Sigma}_{\alpha\gamma}}{\tilde{\Sigma}_{\alpha\beta}}\right)\left(\frac{R_\gamma}{R_0}\right)^2 \frac{1+\cos\gamma}{2}$$
$$- \frac{2\tilde{\Sigma}_{\beta\gamma}}{3\tilde{\Sigma}_{\alpha\beta}}\left(\frac{R_L}{R_0}\right)^2 \frac{R_L}{R_\epsilon}\left[\left(1-\frac{R_\epsilon}{R_L}\right) - \left(\frac{R_\gamma}{R_L}\right)^2 \frac{(1+\cos\gamma)^2}{2}\right]$$
$$+ \frac{2\tilde{\Sigma}_{\beta\gamma}}{3\tilde{\Sigma}_{\alpha\beta}}\left(\frac{R_L}{R_0}\right)^2 \frac{R_L}{R_\epsilon}\left[\frac{R_\epsilon}{R_L}\left(\frac{R_\gamma}{R_L}\right)^3 \frac{2-\cos\gamma}{4}(1+\cos\gamma)^2\right] \quad \text{(S18)}$$
$$- \frac{2\tilde{\Sigma}_{\beta\gamma}}{3\tilde{\Sigma}_{\alpha\beta}}\left(\frac{R_L}{R_0}\right)^2 \frac{R_L}{R_\epsilon}\left[\left(\frac{R_\gamma}{R_L}\right)^4 \cos\gamma\,(1+\cos\gamma)^2\right]$$

We take the limit of a large liposome, where the deformation caused by the droplet leads only to a small change of the liposome radius, $R_\epsilon/R_L \approx 1$, and where the liposome radius is much larger than the curvature radius in the contact region, $R_\gamma/R_L \ll 1$. Eq. S18 thus simplifies to

$$\frac{\Delta E}{4\pi R_0^2 \Sigma_{\alpha\beta}} = \left(\frac{R_\beta}{R_0}\right)^2 \frac{1+\cos\beta}{2} - \left(\frac{\tilde{\Sigma}_{\beta\gamma} - \tilde{\Sigma}_{\alpha\gamma}}{\tilde{\Sigma}_{\alpha\beta}}\right)\left(\frac{R_\gamma}{R_0}\right)^2 \frac{1+\cos\gamma}{2}$$
$$+ \frac{\tilde{\Sigma}_{\beta\gamma}}{3\tilde{\Sigma}_{\alpha\beta}}\left(\frac{R_\gamma}{R_0}\right)^2 (1+\cos\gamma)^2 \left(1+\frac{R_\gamma}{R_L}\left(\frac{2-\cos\gamma}{2}\right)\right) \quad \text{(S19)}$$

A comparison with Eq. S9 shows that the energy of a large liposome can be written in analogy to an infinitely large planar membrane, with $\Sigma_{\alpha\beta} = \tilde{\Sigma}_{\alpha\beta}$, $\Sigma_{\beta\gamma} = \frac{4}{3}\tilde{\Sigma}_{\beta\gamma}$ and $\Sigma_{\alpha\gamma} = \tilde{\Sigma}_{\alpha\gamma} + \frac{1}{3}\tilde{\Sigma}_{\beta\gamma}$. The liposome size alters the scaled membrane tension as:

$$\sigma \to \sigma\left(1+\frac{R_\gamma}{R_L}\left(\frac{2-\cos\gamma}{2}\right)\right) \quad \text{(S20)}$$

Hence, for endocytosis ($R_\gamma = R_0, \gamma = \pi$), we find

$$\sigma \to \sigma\left(1+\frac{R_0}{2R_L}\right) \quad \text{(S21)}$$

5. Supplementary movie captions

Movie S1. This video is a Z-stack rotation along the Y axis, the screen shots are shown in Figure S5 g and h.

Movie S2. We show the zoom in part of the white dashed square area in Figure S5g video, which is extracted from Movie S1.

Movie S3. This video recorded the coacervate droplets spermine/polyU (13/1) that were not engulfed by POPC/DOTAP (20 wt%) liposomes were dissolved and coacervate droplets that were engulfed by liposomes were not dissolved after the addition of 1 µL of 3 M NaCl salt to a total volume of 40 µL of coacervates and liposomes mixture. In this video, the replay speed is 34.5 times faster than the recorded experiment (a time stamp is shown at the bottom left, the total time is 11 min and 23 s).

Movie S4. The process of spermine/polyU (13/1) coacervates (red) go into positively charged POPC/DOTAP (20 wt%) liposomes (green) by endocytosis. This movie is a sequence of images, which were recorded by the SP8X Leica confocal microscope with the 488 and 655 nm laser and show the ATTO-488 and Alexa-647 fluorescence, respectively. In this video, the replay speed is 50 times faster than the recorded experiment (a time stamp is shown at the bottom left, the total time is 20 min and 20 s).

Movie S5. The composition of this video is the same as Movie S4, but the size of the coacervates and liposomes is different from it. In this video, the replay speed is 50 times faster than the recorded experiment (a time stamp is shown at the bottom left, the total time is 2 min and 35 s).

Movie S6. This video was recorded from the same sample as Movie S5, but in a different position, and the size of the coacervates and liposomes is different from Movie S5. In this video, the replay speed is 50 times faster than the recorded experiment (a time stamp is shown at the bottom left, the total time is 22 min and 5 s).

References

(1) Lu, T.; Spruijt, E. Multiphase Complex Coacervate Droplets. *J. Am. Chem. Soc.* **2020**, *142* (6), 2905-2914. DOI: 10.1021/jacs.9b11468.

(2) Wang, F.; Zhang, D.; Duan, C.; Jia, L.; Feng, F.; Liu, Y.; Wang, Y.; Hao, L.; Zhang, Q. Preparation and characterizations of a novel deoxycholic acid–O-carboxymethylated chitosan–folic acid conjugates and self-aggregates. *Carbohydr. Polym.* **2011**, *84* (3), 1192-1200. DOI: 10.1016/j.carbpol.2011.01.017.

(3) Tsugane, M.; Suzuki, H. Elucidating the Membrane Dynamics and Encapsulation Mechanism of Large DNA Molecules Under Molecular Crowding Conditions Using Giant Unilamellar Vesicles. *ACS synth. biol.* **2020**, *9* (10), 2819-2827. DOI: 10.1021/acssynbio.0c00360.

(4) Lu, T.; Nakashima, K. K.; Spruijt, E. Temperature-Responsive Peptide-Nucleotide Coacervates. *J. Phys. Chem. B* **2021**, *125* (12), 3080-3091. DOI: 10.1021/acs.jpcb.0c10839.

(5) Kurniawan, J.; Ventrici de Souza, J. F.; Dang, A. T.; Liu, G. Y.; Kuhl, T. L. Preparation and Characterization of Solid-Supported Lipid Bilayers Formed by Langmuir-Blodgett Deposition: A Tutorial. *Langmuir* **2018**, *34* (51), 15622-15639. DOI: 10.1021/acs.langmuir.8b03504.

(6) Buahom, P. Measuring the Contact Angle in ImageJ (with the plug-in Contact Angle). **2018**.

(7) Kusumaatmaja, H.; Lipowsky, R. Droplet-induced budding transitions of membranes. *Soft Matter* **2011**, *7* (15), 6914. DOI: 10.1039/c1sm05499f.
